# Curaxin CBL0137 Exerts Anticancer Activity via Diverse Mechanisms

**DOI:** 10.3389/fonc.2018.00598

**Published:** 2018-12-07

**Authors:** Ming-Zhu Jin, Bai-Rong Xia, Yu Xu, Wei-Lin Jin

**Affiliations:** ^1^Shanghai Jiao Tong University School of Medicine, Shanghai, China; ^2^Department of Gynecology, The Affiliated Tumor Hospital, Harbin Medical University, Harbin, China; ^3^Key Laboratory for Thin Film and Microfabrication Technology of Ministry of Education, Department of Instrument Science and Engineering, Shanghai Engineering Center for Intelligent Diagnosis and Treatment Instrument, School of Electronic Information and Electronic Engineering, Institute of Nano Biomedicine and Engineering, Shanghai Jiao Tong University, Shanghai, China; ^4^National Center for Translational Medicine, Collaborative Innovational Center for System Biology, Shanghai Jiao Tong University, Shanghai, China; ^5^Shaanxi Key Laboratory of Brain Disorders and Institute of Basic and Translational Medicine, Xi'an Medical University, Xi'an, China

**Keywords:** cancer stem cells, CBL0137, chemotherapy, facilitates chromatin transcription, p53

## Abstract

Chemotherapy with or without radiation remains the first choice for most cancers. However, intolerant side effects and conventional drug resistance restrict actual clinical efficacy. Curaxin CBL0137 is designed to regulate p53 and nuclear factor-κB simultaneously and to prevent the resistance caused by a single target. Functionally, CBL0137 exhibits an antitumor activity in multiple cancers, including glioblastoma, renal cell carcinoma, melanoma, neuroblastoma, and small cell lung cancer (SCLC). Mechanistically, CBL0137 is originally identified to act by facilitates chromatin transcription (FACT) complex. Further investigations reveal that several pathways, such as NOTCH1 and heat shock factor 1 (HSF1), are involved in the process. CBL0137 has been reported to target cancer stem cells (CSCs) and enhance chemotherapy/monotherapy efficacy. The translational advance of CBL0137 into clinical practice is expected to provide a promising future for cancer treatment.

## Introduction

Cancer harbors several characteristics, including high heterogeneity, diverse gene mutation, or rapid progression; consequently, treating cancer is difficult, and it easily relapses. Remarkable achievements have been observed in treatment approaches, including surgery, radiotherapy, chemotherapy, immunotherapy, and targeted therapy. In particular, targeted therapies, such as HER2 inhibitor lapatinib, EGFR inhibitor erlotinib, BRAF inhibitor dabrafenib, promote treatment (1). However, we have failed to treat cancer. Malignancies, such as glioblastoma, are quite invasive and cannot be entirely removed by surgery. Chemotherapy is hindered by innate and acquired chemoresistance.

Originally, antimalarial agents, including quinacrine, can activate p53 and inhibit nuclear factor-κB (NF-κB) simultaneously (2, 3). These drugs have been used as a reference of curaxins, undergoing some structural changes but maintaining similar functions (2, 4). As a second-generation curaxin, CBL0137 satisfies the requirements for a drug design, that is, full efficacy while inducing the least adverse effects. Further research suggested that CBL0137 exerts an antitumor activity through multiple targets, including facilitates chromatin transcription (FACT), NOTCH1, and heat shock factor 1 (HSF1), in various cancers (Table 1). At present, CBL0137 in patients with hematological malignancies (ClinicalTrials.gov Identifier: NCT02931110) and solid tumors are under phase I clinical trials (ClinicalTrials.gov Identifier: NCT01905228). In this review, we summarized the design of CBL0137, highlighted its antitumor mechanisms through multiple targets, and proposed its potential for clinical applications, especially as a combination drug.

**Table 1:** Targets and induced effects of CBL0137 reported in cancer research.

**Indications**	**Targets**	**Effects**	**Experiment models**	**References**
Glioblastoma	SSRP1↓ SOX2↓ OCT4↓ NANOG↓ OLIG2↓ CD133↓	Inhibited proliferation of patient-derived tumor cells	Cell lines Orthotopic mouse models	(5)
Glioblastoma (2)	FACT↓ p53↑ NF-κB↓	Induced Apoptosis and inhibited proliferation Increased survival of TMZ-responsive and -resistant GBM	Cell lines Orthotopic mouse models	(6)
Renal cell carcinoma	p53↑ NF-κB↓	Induced death of tumor cells through FACT with no DNA damage	Cell lines PDX mouse models	(7)
Melanoma	p53↑ NF-κB↓ HSF1↓	Enhanced anti-tumor activity by inhibiting heat shock responses of tumor cells	Cell lines Orthotopic mouse models	(8)
Neuroblastoma	MYCN↓	Reduced tumor initiation and progression	Cell lines TH-MYCN transgenic mouse models	(9)
Neuroblastoma (2)	SSRP1↓ SPT16↓ MYCN↓	Inhibited neuroblastoma cell growth	MYCN transgenic zebrafish	(10)
Small cell lung cancer	NOTCH1↑	Reduced the tumor cell growth Preferentially kills tumor-initiating cells	Cell lines PDX mouse models	(11)

*PDX, patient-derived xenograft*.

## CBL0137: a Second-Generation Curaxin

Small molecular inhibitor CBL0137 [1,1′-(9-(2-(isopropylamino)ethyl)-9H-carbazole-3,6-diyl)bis(ethan-1-one) (IUPAC/chemical name)] is a second-generation curaxin. Dermawan et al. (12) found that quinacrine (CBLC-102), a first-generation curaxin, can overcome erlotinib resistance through preconceived mechanisms in non-small cell lung cancer (12). Similar results are also observed in ovarian (13) and breast (14) cancers. Second-generation curaxins, such as CBLC-000, CBLC-100, and CBLC-137 (CBL0137), have more exact targets than first-generation curaxins, such as quinacrine (CBLC-102). In particular, CBL0137 is water soluble because of its chemical structure and better tolerated in mice than other members of curaxins, showing great potential for cancer treatment (2). In addition to the two targets, namely, p53 and NF-κB, CBL0137 can intercalate DNA through FACT without causing any DNA damage or genotoxicity (2, 7, 15), and more targets are under investigation.

## FACT: A Core Target For CBL0137

**Figure 1:**
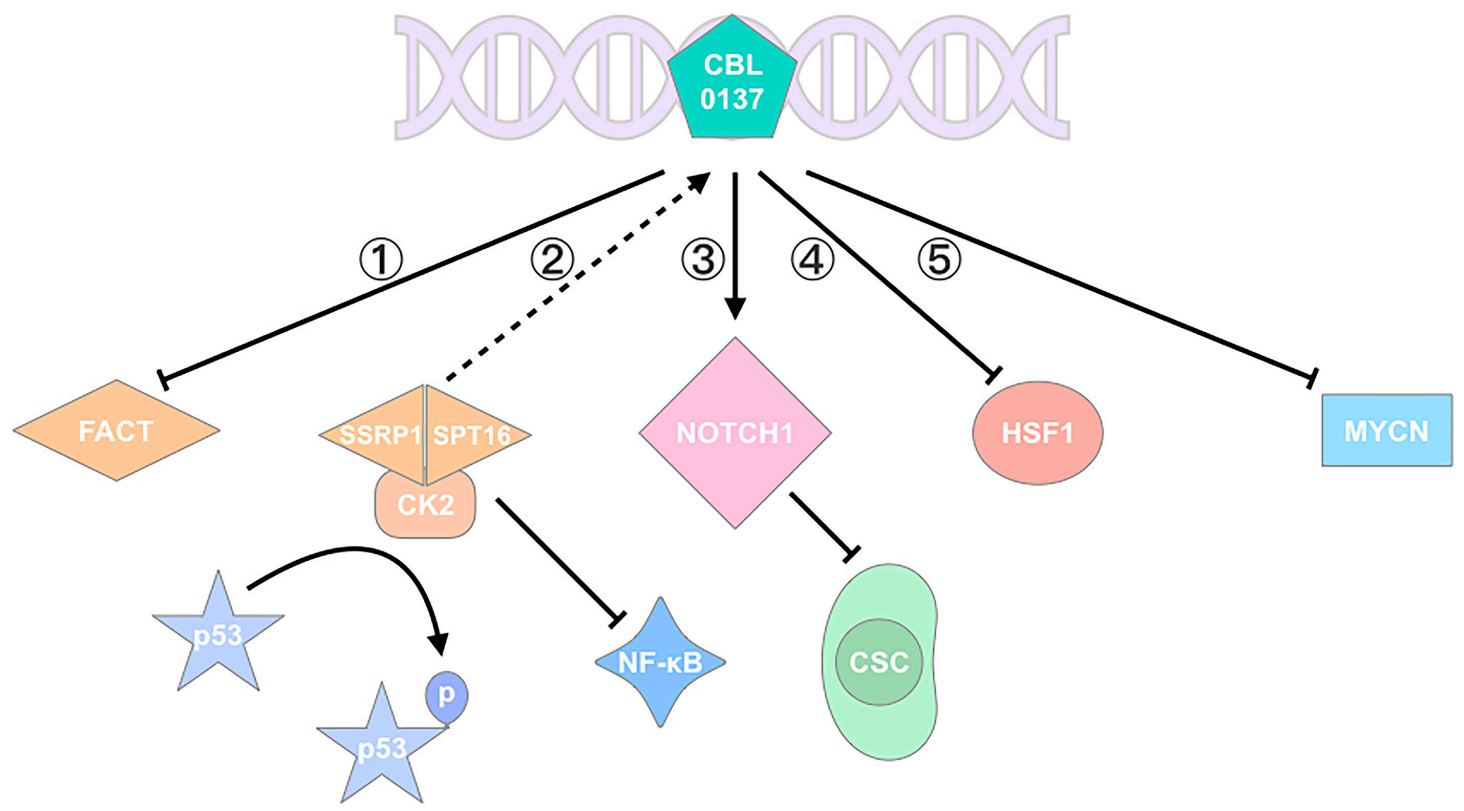
CBL0137 exhibits its antitumor activity via multiple pathways. (1) CBL0137 can target SPT16 and SSRP1, the two subunits of FACT; (2) CBL0137 can induce p53 activation by phosphorylation and NF-κB inhibition, depending on the formation of SPT16-SSRP1-CK2 complex; (3) CBL0137 can reduce the self-renewal of CSCs through NOTCH1 signaling pathway activation; (4) CBL0137 can decrease HSF1 transcription level; (5) CBL0137 can downregulate MYCN expression.

FACT, a histone chaperone, contains two subunits of the suppressor of Ty 16 (SPT16) and structure-specific recognition protein 1 (SSRP1), which participates in DNA replication, transcription, repair, mitosis, and cell fate reprogramming (16–18). SPT16 remodels the histone structure after transcription, and SSRP1 recognizes nucleosomes with its high-mobility group (HMG)-1 domain (19, 20). SSRP1 is considered more like a target since it's more amplified in cancers at mRNA and protein levels. FACT is involved In the poor prognosis, malignant transformation, tumorigenesis, and aggressiveness of cancers (9, 16, 21–23). It can recognize the formation of alternative DNA structures and promote the activation of p53 to prevent DNA damage (24); thus FACT is regarded as a sensor for genome instability and mutation, which is one of the ten hallmarks of cancer treatment (24, 25) (Figure 1). It is highly expressed in cancer including glioblastoma (GBM) (6), breast cancer (16), and hepatocellular carcinoma (21), but is poor expressed in normal tissues or well-differentiated cells (26).

CBL0137, chemotherapeutic agents, UV radiation, oxygen-free radicals, and hypoxia stress can affect p53 activation (7, 27, 28). With Western Blot analysis, Gasparian et al. (7) have revealed that CBL0137 activates p53 through posttranslational modifications at serine 392 (Ser^392^) rather than serine 15 (Ser^15^), which involves casein kinase 2 (CK2) inhibition (7). Previous studies showed that CK2-induced p53 phosphorylation involves FACT. FACT, SPT16, and SSRP1 subunits, can bind to CK2 after CBL0137 is administered, and the SPT16-SSRP1-CK2 complex phosphorylates p53 at Ser^392^ and promotes p53 activation (2, 27) (Figure 1). Activated p53 induces apoptosis, promotes DNA repair, and inhibits tumor growth. Extensive evidence has also demonstrated that FACT can promote tumor growth, inhibit apoptosis or cell differentiation and induce cell proliferations through the regulation of multiple genes including TP53, MYC, NF-κB, OCT1, and HSF1 (23) (Figure 1).

FACT has recently been reported to correlate with the expression of cancer stem cell (CSC) markers, such as SOX2, OCT4, OLIG2, and NANOG in an adult GBM model. The transcriptional knockdown of FACT or its inhibition with a small molecule (CBL0137) reduces the expression of these genes (5).

## CBL0137 Exerts Antitumor Activity by Increasing p53 and Decreasing NF-κB Simultaneously

p53 is a classic tumor suppressor protein responsible for the prevention of oncogenic mutation accumulation, tumorigenesis and tumor progression (29). p53 mutation or inactivation is quite common in many cancers (30). p53 activities are regulated by diverse post-translational modifications such as Ser^15^ and Ser^392^ phosphorylation or lysine 382 acetylation and methylation (7, 31). NF-κB is a critical transcription factor in antiapoptosis and cell proliferation, which is activated in inflammation and cancers (32). CBL0137 is originally designed to activate p53 and inhibit NF-κB simultaneously to achieve an enhanced efficacy with modest toxicity (7).

The *in vitro* and *in vivo* experiments of CBL0137 have confirmed the issue. For example, a research on renal cell carcinoma has suggested that CBL0137 intercalates DNA and traps FACT, thereby leading to NF-κB inhibition. FACT binds to CK2 to form a complex, which further induces Ser^392^ phosphorylation of p53; otherwise, p53 is degraded by MDM2 (2, 7, 33) (Figure 1). Meanwhile, NF-κB is inhibited by the complex (6). Similar results have been shown in GBM research, temozolomide (TMZ)-resistant A1207, TMZ-responsive U87MG cell lines, and orthotopic model. CBL0137 prolongs the survival of orthotopic A1207 and U87MG models, though it is less effective than TMZ in the latter. Furthermore, 0.6 and 2.0 μM CBL0137 can increase p53 significantly in cell lines. These studies have exhibited the antitumor activity of CBL0137 by targeting p53 and NF-κB, which are the two most common transcription factors in oncogenic and tumor suppressor pathways.

## CBL0137 Inhibits the Self-Renewal of Cancer Stem Cells/Tumor-Initiating Cells Through NOTCH1 Activation

Therapeutic resistance is a complex phenomenon in cancer treatment, though many mechanisms have been proposed. “The bad seed” CSCs can explain the consequence to some degree (34). Conventional therapies that do not target CSCs may encounter cancer recurrence because CSCs can undergo self-renewal and differentiation (35). Dermawan et al. investigated CBL0137 in GBM and focused on cancer stem-like cells by using CD133 as a marker (5). CBL0137 accumulates in brain tissues in orthotopic mouse models, suggesting that it can penetrate the blood brain barrier; oral intake *ad libitum* can also achieve its efficacy. CBL0137 prefers to inhibit CD133+ tumor cell growth with the help of FACT, which is higher in CSCs than non-stem tumor cells. CBL0137 treatment decreases the expression of CD133 and the self-renewal of CSCs, increases asymmetric cell division, prevents tumor initiation and prolongs the survival of tumor-bearing animals (5). A similar consequence has been demonstrated in small cell lung cancer (SCLC) and pancreatic cancer (11, 36). Tumor-initiating cells (TICs) represent those with stemness. CBL0137 preferentially reduces CD133^high^ and CD44^high^ cells (TICs) over CD133^low^ and CD44^low^ (non-TICs) and attenuate the self-renewal of TICs (11).

The stemness of CSCs is well-modulated by stem-cell factors including p53, NF-κB, Sox2, Bmi1, c-Myc, and NOTCH1 (35, 37). Therefore, drugs should target CSCs and CSC-related factors. NOTCH signaling pathway plays a role in oncogenesis, angiogenesis and CSC maintenance (38). It exhibits oncogenic and suppressive roles in different cancers (11). NOTCH1, as a member of the NOTCH family, increases apoptosis and inhibits cell proliferation in SCLC (11). CBL0137 treatment in SCLC prevents SP3 binding to the NOTCH1 promoter, decreases achaete-scute homolog-1 (ASCL1) expression, increases the mRNA expression of NOTCH1, and inhibits CSC renewal. The expression levels of ASCL1 and SP3 are higher in TICs than non-TICs, negatively modulating NOTCH1. Therefore, the tendency of CBL0137 killing TICs may be a result of FACT and NOTCH1, though whether CBL0137 targeting NOTCH1 acts through FACT is unclear in this research (11). CBL0137 can activate NOTCH1 and inhibit the self-renewal of CSCs/TICs (5, 11, 36), thereby facilitating the enhanced prevention of therapeutic resistance and tumor progression.

## HSF1 Is Involved in the Antimelanoma Effect of CBL0137

Regional chemotherapy via isolated limb perfusion (ILP) is recommended for patients with in-transit extremity melanoma in which mild hyperthermia (42°C compared with 37°C) is adopted, thereby improving drug uptake by tumor cells (8). CBL0137 was then tested for potential use as a regional chemotherapeutic agent on B16 melanoma cell line and tumor-bearing mice. CBL0137 treatment by ILP reduces SSRP1 expression, suppresses HSF1/hsp70 transcription, and causes tumor cell death, and its efficacy can be improved by hyperthermia. Conversely, CBL0137 can downregulate HSF1 to inhibit heat shock responses brought by hyperthermia, thereby increasing tumor cell apoptosis. However, treatment of traditional melphalan had no statistically significant differences between 42 and 37°C. Moreover, the linkage of the ILP drug melphalan can be highly toxic and cause death. By contrast, even 0.1 mg of CBL0137 establishes a strong antitumor activity, suggesting its leakage causes minimal side effects (8). These results explain the antitumor mechanism of CBL0137 from the perspective of hyperthermia and HSF1, suggesting that CBL0137 can be considered as a promising candidate for ILP drug to treat melanoma.

## MYCN in Neuroblastoma: A Potential Indicator of CBL0137 Sensitivity

Approximately 20% of patients with neuroblastoma encounter MYCN amplification, which is a predictor of poor prognosis (9, 39). Considering that the expression of FACT and MYCN is closely related and high in precancerous TH-MYCN^+/+^ neuroblasts, Carter et al. (9) treated TH-MYCN^+/+^ and TH-MYCN^+/−^ mice with CBL0137, which is regarded as the inhibitor of FACT. CBL0137 can downregulate FACT and MYCN expression and inhibit MYCN-driven tumor initiation and progression in MYCN mice and xenografts. In tumor-bearing zebrafish, CBL0137 elicits an inhibitory effect on neuroblastoma (10). Moreover, high-MYCN-expressing cell lines, such as SH-SY5Y and BE(2)C, require a lower IC_50_ of CBL0137 than those expressing normal or relatively low MYCN, suggesting that MYCN expression may be applied to evaluate CBL0137 sensitivity, though further investigation is needed (9).

## Combination Approach of CBL0137: The Way to Go

The initial goal of scientists from Clevel and BioLabs Inc. in designing curaxins is to regulate p53 and NF-κB (2). After the “target multiplier” FACT is introduced, the understanding of curaxins has improved. CBL0137 can reduce CSC populations and their stemness (5, 11, 36), which show its promising clinical prospect combined with standard treatment strategies.

The cisplatin resistance of SCLC is likely caused by CSCs. In this research, the combination of CBL0137 and cisplatin at a 1:1 molar ratio remarkably inhibits SCLC tumor growth in H82 xenograft (11). Drug combination delays tumor growth for 30 days and prolongs tumor-bearing mice survival for more than 10 days (11). FACT plays an important role in DNA repair; thus, researchers believed that these results may be due to FACT and its ability to inhibit DNA repair, though this hypothesis has yet to be further investigated (11). However, this hypothesis is partially confirmed in neuroblastoma. Combined with cisplatin, cyclophosphamide, etoposide, or vincristine, CBL0137 can inhibit DNA repair after a double-strand break occurs without genotoxicity. DNA damage markers remarkably increase after etoposide and CBL0137 are administered. The results showed that the effects of CBL0137 are observed in DNA synthesis inhibitors, such as hydroxyurea, rather than microtubule poisons, such as hydroxyurea (9). Another research has shown that the combination of CBL0137 and TMZ does not significantly affect GBM. Combination therapy surpasses CBL0137 monotherapy but not that of TMZ (6). These results are not satisfactory for GBM, but they provide insights into CBL0137 combined with chemotherapy. Further research should be conducted on this area.

Early studies revealed the crosstalk between NF-κB and epidermal growth factor receptor (EGFR), describing them as “partners in cancer” (31, 40–44). In a GBM research, EGFR inhibitor lapatinib and CBL0137 are combined at a 10:1 molar ratio. Lapatinib seldom inhibits CSC growth, which partially explains why it fails to achieve a satisfactory clinical efficacy in GBM treatment (5). The combination of lapatinib and CBL0137 confirms Shostak and Chariot's outlook and presents possibilities for CBL0137 to be applied with targeted therapy.

## Conclusions and Further Directions

Various small molecules, including PRIMA-1, COTI-2, ReACp53, ZMC1, PK7088 (45–51), and CBL0137, target p53 and have been at preclinical and clinical stages. CBL0137 has a broad antitumor activity in a wide range of cancers, other than targeting p53 (7). CBL0137 can be considered as a candidate for monotherapy and applied to enhance the effectiveness of chemotherapy and targeted therapy, giving it more potential and clinical significance.

However, some concerns still exist. Tumor suppressor protein p53 is important in the oncogenic pathway, and almost 50% of cancers possess mutated or depleted p53; thus, resistance likely exists when one path is blocked. *In vitro* data have also shown that p53-wild type cells are slightly more susceptible to curaxins, including CBL0137-induced cell death, than p53-null cells (7). Discovering how CBL0137 works on those cancers is quite important; in addition, the effect of CBL0137 on the immune system is unknown, and further data support should be obtained to determine whether CBL0137 can synergize with immunotherapy to provide an enhanced efficacy. Further studies on these areas may lead to an in-depth understanding of the mechanism and application of CBL0137.

## Author Contributions

M-ZJ and B-RX conceived, wrote the manuscript, and completed the figure/table. YX contributed to the writing. W-LJ conceived, organized, and edited the text.

### Conflict of Interest Statement

The authors declare that the research was conducted in the absence of any commercial or financial relationships that could be construed as a potential conflict of interest.
